# Evaluating the efficacy of leading large language models in the Japanese national dental hygienist examination: A comparative analysis of ChatGPT, Bard, and Bing Chat

**DOI:** 10.1016/j.jds.2024.02.019

**Published:** 2024-02-29

**Authors:** Shino Yamaguchi, Masaki Morishita, Hikaru Fukuda, Kosuke Muraoka, Taiji Nakamura, Izumi Yoshioka, Inho Soh, Kentaro Ono, Shuji Awano

**Affiliations:** a School of Oral Health Sciences, Kyushu Dental University, Kitakyushu, Japan; b Division of Clinical Education Development and Research, Department of Oral Function, Kyushu Dental University, Kitakyushu, Japan; c Health Information Management Office, Kyushu Dental University Hospital, Kitakyushu, Japan; d Division of Maxillofacial Surgery, Department of Physical Function, Kyushu Dental University, Kitakyushu, Japan; e Division of Periodontology, Department of Oral Function, Kyushu Dental University, Kitakyushu, Japan; f Division of Oral Medicine, Department of Physical Function, Kitakyushu, Japan; g Division of Physiology, Department of Health Promotion, Kyushu Dental University, Kitakyushu, Japan

**Keywords:** Education tool, GPT-4, Japanese national dental hygienist examination, Large language models

## Abstract

**Background/purpose:**

Large language models (LLMs) such as OpenAI's ChatGPT, Google's Bard, and Microsoft's Bing Chat have shown potential as educational tools in the medical and dental fields. This study evaluated their effectiveness using questions from the Japanese national dental hygienist examination, focusing on textual information only.

**Materials and methods:**

We analyzed 73 questions from the 32nd Japanese national dental hygienist examination, conducted in March 2023, using LLMs ChatGPT-3.5, GPT-4, Bard, and Bing Chat. Each question was categorized into one of nine domains. Standardized prompts were used for all LLMs, and Fisher's exact test was applied for statistical analysis.

**Results:**

GPT-4 achieved the highest accuracy (75.3%), followed by Bing (68.5%), Bard (66.7%), and GPT-3.5 (63.0%). There were no statistically significant differences between the LLMs. The performance varied across different question categories, with all models excelling in the ‘Disease mechanism and promotion of recovery process' category (100% accuracy). GPT-4 generally outperformed other models, especially in multi-answer questions.

**Conclusion:**

GPT-4 demonstrated the highest overall accuracy among the LLMs tested, indicating its superior potential as an educational support tool in dental hygiene studies. The study highlights the varied performance of different LLMs across various question categories. While GPT-4 is currently the most effective, the capabilities of LLMs in educational settings are subject to continual change and improvement.

## Introduction

It is well known that ChatGPT and other large language models (LLMs) are rapidly innovating and improving. Commonly known LLMs include ChatGPT, Bard, and BingChat (developed by OpenAI, Google, and Microsoft, respectively). These models have advanced conversational abilities, closely resembling human-like interactions. This capability holds excellent promise for educational settings, including the use of virtual assistants, chatbots, and online learning support systems.^1^ Evaluations of correct response rates using LLMs have been reported in national examinations for medical doctors, nurses, and pharmacists, suggesting the potential of LLMs as an educational support tool.^2^, ^3^, ^4^

ChatGPT-4 has been reported to have statistically significantly more dental knowledge than GPT-3.5.^5^ These reports were based on evaluations using ChatGPT-3.5 or GPT-4, focusing specifically on questions with textual information, excluding figures, tables, and image information.^2^, ^3^, ^4^, ^5^ ChatGPT and other LLMs have been evaluated in previous reports in the medical and dental field.^2^, ^3^, ^4^, ^5^, ^6^, ^7^, ^8^, ^9^, ^10^ However, the Japanese national dental hygienist examination has not yet been evaluated by any LLMs.

Therefore, the present study was conducted as a pilot study using multiple LLMs to clarify the potential of LLMs as educational support tools, using questions from the Japanese national dental hygienist examination and targeting only questions with textual information, excluding charts and images.

## Materials and methods

### Obtaining and processing data from the Japanese national dental hygienist examination

We collected questions from the 32nd Japanese national dental hygienist examination, administered in March 2023, using the 32nd national dental hygienist examination question booklet.^11^ The national dental hygienist examination consists of 220 questions, and in the present study, only questions that did not include figures or images were extracted; 74 questions were selected. The Ministry of Health, Labour and Welfare in Japan evaluates examination questions after they are administered and publishes on the web the questions eliminated from scoring as inappropriate.^12^ Of the 74 questions, one was excluded as inappropriate; 73 were used in this study.

The Guideline for National Dental Hygienist Examination divides the examination subjects into the following nine categories: structure and function of the human body excluding teeth and oral cavity, structure and function of teeth and oral cavity, disease mechanism and promotion of recovery process, human and social systems related to dental and oral health and prevention, introduction to dental hygiene, clinical dental medicine, theory of preventive dental procedures, theory of dental health guidance, and theory of dental assistance.^13^ Using the 32nd national dental hygienist examination Question Booklet, each question was identified as belonging to one of these nine categories.^11^

### Large language models

ChatGPT-3.5 and 4, developed by OpenAI (September 2023 model), Bard, developed by Google (19 September 2023 model), and Bing Chat, developed by Microsoft (GPT-4 based model), were used as LLMs. Standardized prompt inputs and question texts were entered into each LLM's web interface, and all responses obtained were recorded. The input was performed on 12 November 2023. A prompt is “an instruction given to an LLM to enforce a rule, automate a process, or guarantee a specific quality and quantity of the generated output”.^14^

We standardized the format of the prompts when entering the question text and images as follows: “You are a student taking the National Dental Hygienist Examination. According to the question text, first output the correct answer. Then, output the reason for your choice and why the other choices are inappropriate.” In this study, to ensure consistency, each prompt and question text entry into the LLMs was attempted only once per question, and each response was recorded.

**Figure 1:**
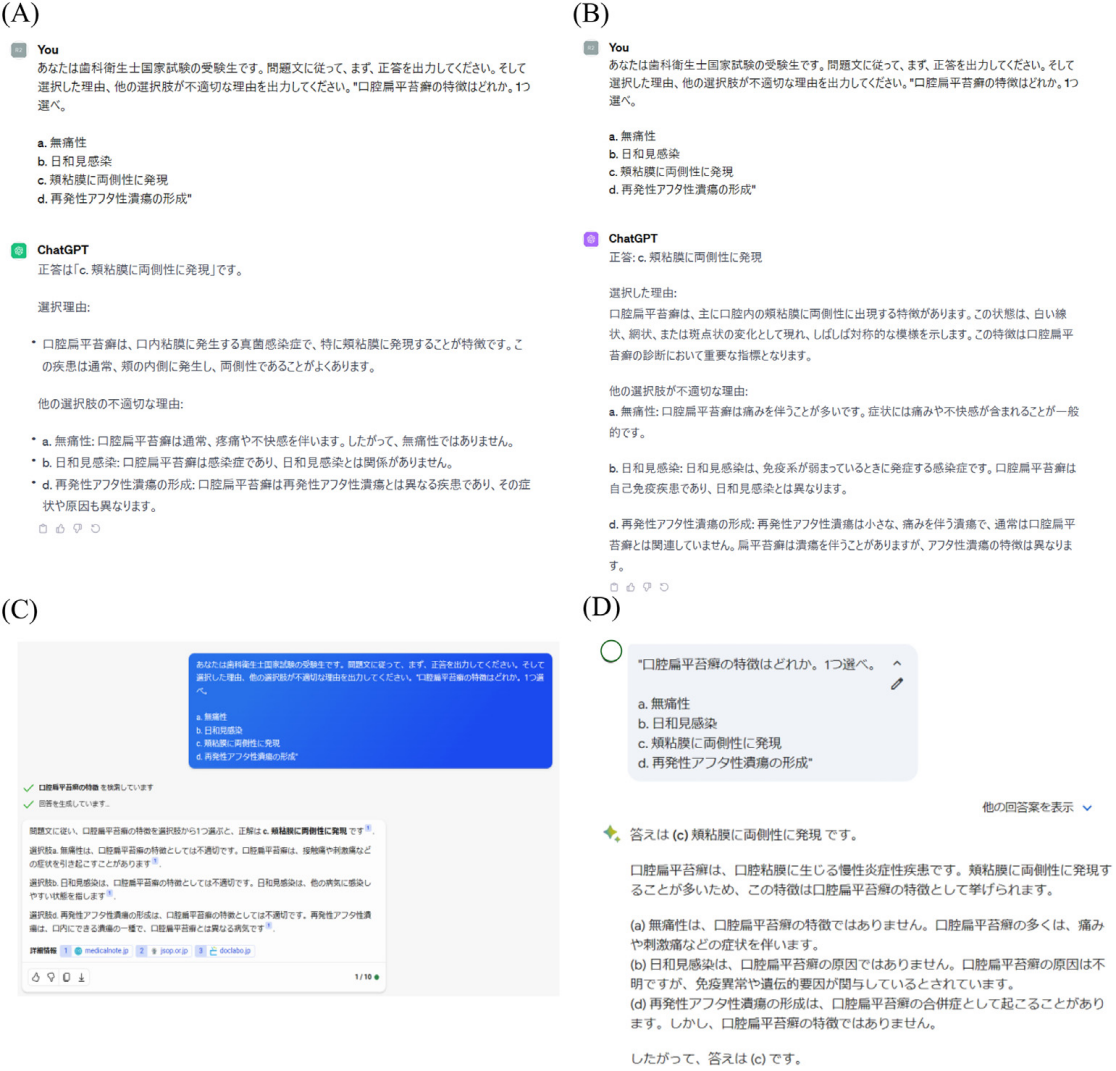
Images of the prompt and question text inputted into each large language model (LLM), (A) ChatGPT-3.5, (B) ChatGPT-4, (C) Bing Chat, (D) Bard.

Figure 1 shows the images of the prompt and question text inputted into each LLM. Specifically, Fig. 1A illustrates the input for ChatGPT-3.5, Fig. 1B for ChatGPT-4, Fig. 1C for Bing Chat, and Fig. 1D for Bard.

### Data and statistical analysis

We used QlikSense® Enterprise August 2022 Patch 2 (Qlik Technologies Inc., King of Prussia, PA, USA) for data analysis. We used GraphPad Prism 9.5.1 (GraphPad Software, Boston, MA, USA) for statistical analysis employing Fisher's exact test.

## Results

Table 1 shows the results of the questions using each LLM. There was one unanswerable question in Bard. The highest percentage of correct answers was 75.3% for GPT-4, 68.5% for Bing, 66.7% for Bard, and 63.0% for GPT-3.5. Fisher's exact test was performed between each LLM and no statistically significant difference was found between combinations.

**Table 1:** Performance of large language models (LLMs) in the Japanese National Dental Hygienist Examination.

LLMs	Number of questions (n)	Correct answers (n)	Incorrect answers (n)	Percentage of correct answers (%)
GPT3.5	73	46	27	63.0
GPT4	73	55	18	75.3
Bard	72	48	24	66.7
Bing	73	50	23	68.5

Table 2 shows the percentage of correct answers and other data by the number of correct answers specified in the question text. When the number of correct answers specified by the question text was 1, the LLMs showed more than 72.5% of correct answers, except for Bing, which was 68.3%. When the number of correct responses was 2, the percentages of correct answers were 50.0% for GPT-3.5 and 59.4% for Bard, but 68.8% for Bing and 75.0% for GPT-4. Fisher's exact test was used to evaluate between LLMs, but none of the differences were statistically significant.

**Table 2:** Comparative performance of large language models (LLMs) based on the number of correct answers.

Number of specified correct answers	LLMs	Correct answers (n)	Incorrect answers (n)	Percentage of correct answers (%)
1	GPT3.5	30	11	73.2
	GPT4	31	10	75.6
	Bard	29	11	72.5
	Bing	28	13	68.3
2	GPT3.5	16	16	50.0
	GPT4	24	8	75.0
	Bard	19	13	59.4
	Bing	22	10	68.8

Table 3 shows the accuracy of the four LLMs' answers to questions related to each category of the exam, rated as a percentage of correct answers. In theory of dental health guidance, GPT-3.5, Bard, and Bing exhibited similar accuracy (73.3%, 73.3%, and 80.0%, respectively) for 15 questions. GPT-4 outperformed the other models with 86.7%. In human and social systems related to dental and oral health and prevention, all models demonstrated comparable performance, scoring 71.4%, except GPT-3.5 (64.3%). In theory of preventive dental procedures, the models faced 11 questions, with GPT-4 and Bard having a 54.5% accuracy rate, lower than Bing and GPT-3.5 (both 63.6%). For the 10 questions in theory of dental assistance, GPT-3.5 scored 50.0%, Bard scored 60.0%, Bing scored 70.0%, and GPT-4 scored 80.0%. In clinical dental medicine, among the nine questions posed, GPT-4 scored highest with 77.8%, followed by Bard and Bing with 55.6%, and GPT-3.5 with 44.4%. For the five questions in introduction to dental hygiene, Bing and GPT-3.5 both scored 80.0% while GPT-4 and Bard both achieved 60.0%. In structure and function of teeth and oral cavity, with three questions, GPT-4 scored 66.7%, GPT-3.5 and Bard scored 33.3%. Bing did not score in this category. In the 3-question category of disease mechanism and promotion of recovery process, all models performed exceptionally well, each achieving 100.0%. In structure and function of the human body excluding teeth and oral cavity, GPT-3.5 and Bing scored 66.7% for the three questions, while GPT-4 and Bard achieved a perfect score of 100.0%.

**Table 3:** Performance of large language models (LLMs) in different categories of the Japanese National Dental Hygienist Examination

Category	Number of questions (n)	Percentage of correct answers (%)			
		GPT3.5	GPT4	Bard	Bing
Theory of dental health guidance	15	73.3	86.7	73.3	80.0
Human and social systems related to dental and oral health and prevention	14	64.3	71.4	71.4	71.4
Theory of preventive dental procedures	11	63.6	54.5	54.5	63.6
Theory of dental assistance	10	50.0	80.0	60.0	70.0
Clinical dental medicine	9	44.4	77.8	55.6	55.6
Introduction to dental hygiene	5	80.0	60.0	60.0	80.0
Structure and function of teeth and oral cavity	3	33.3	66.7	33.3	0.0
Disease mechanism and promotion of recovery process	3	100.0	100.0	100.0	100.0
Structure and function of the human body excluding teeth and oral cavity	3	66.7	100.0	100.0	66.7
Total	73	63.0	75.3	65.8	68.5

The total data set consisted of 73 questions, and the overall accuracy was, in order of highest to lowest, GPT-4 (75.3%), Bing (68.5%), Bard (65.8%), and GPT-3.5 (63.0%).

## Discussion

We used four LLMs, GPT-3.5, GPT-4, Bard, and Bing, to evaluate the ability to pass questions on the Japanese national dental hygienist examination that did not include charts or intraoral photographs. Our results showed that GPT-4 had the highest percentage of correct answers among the four LLMs, consistent with a previous study comparing GPT-3.5 and GPT-4 on dental knowledge.^5^ The results were also consistent with those of a report evaluating the percentage of correct answers on GPT-3.5 and GPT-4 in national examinations in the medical field.^2^, ^3^, ^4^

Our evaluation using the 73 questions in our study showed that the highest correct response rates were for GPT-4, followed by Bing, Bard, and GPT-3.5. However, no statistically significant differences were found for any combination of the four LLMs. Bing had the second-highest percentage of correct answers after GPT-4, possibly because Bing's operation is based on GPT-4.^15^ However, it is clear from the present study that the results differed from those of GPT-4. Although one report claimed that Bing Chat had a higher percentage of correct responses than GPT-4, the results may have differed because that study used a data set of English questions, which was entirely different from ours.^6^ It is generally expected that results differ depending on the data set.

Bard gave no answer to one question, which we treated as unanswerable; we entered the same question into Bard multiple times, only to repeatedly get the same answer of being unable to give a correct answer. The behavior was different depending on the LLMs in the present study. A report evaluating the performance of GPT-3.5, GPT-4, and Bard on the neurosurgery oral exam prep question bank showed that GPT-4 outperformed both GPT-3.5 and Bard, consistent with our finding that GPT-4 is most effective.^16^

When the number of correct answers indicated by the question was 1, the correct response rate was 70%, except for Bing, indicating that the question was easy for the LLMs. When the number of correct answers indicated by the question was 2, GPT-4 had the highest correct response rate of 75.0%, demonstrating the superior ability of GPT-4. Furthermore, the percentage of correct LLMs varied depending on the instructions for the number of correct answers.

The Japanese national dental hygienist examination is classified into nine categories, and Table 3 shows the differences in the correct response rates of the LLMs according to category. In some cases, the correct response rates of the LLMs differed significantly from category to category, while in others, such as the disease mechanism and promotion of recovery process category, all LLMs showed 100% correct response rates. The difference in the percentage of correct responses by each LLM may be partly due to differences in the training data of each LLM.

As shown in Table 3, GPT-4 generally had the highest percentage of correct responses, again demonstrating, as in Table 2, the high ability of GPT-4. Other reports similarly showed high GPT-4 capacity, consistent with the results of this study. However, in some categories, the correct answer rate of GPT-4 was lower than that of other LLMs, and it was found that it was weak in theory of preventive dental procedures and introduction to dental hygiene. It is essential to note that while LLMs have shown promise in various applications, they also present challenges. For instance, they can generate false, erroneous, or misleading content, a significant concern in examinations and educational settings.^17^

The primary aim of this study was to evaluate the correct answer rates of four LLMs and their problem-solving abilities. Additionally, the LLMs were instructed to provide the rationale behind their answers. However, this aspect was not the focus of our analysis. Therefore, the study concentrated solely on assessing the percentage of correct answers without exploring the underlying reasoning that LLMs articulated for their answers. Although this approach was methodologically intentional, we recognize that this is a limitation of the study. Future research is envisaged to conduct a comprehensive analysis encompassing both the accuracy of the responses and the substantiation of the responses provided by the LLMs.

In this study, we evaluated only once trial. We recognize that the number of trials may influence the study's results, and this is one of the limitations of this study. Looking forward, we aim to extend our inquiry to understand how the variability in the number of trials could affect the accuracy of the response rates from the LLMs.

Another limitation of this study is that although there are studies comparing GPT-3.5 to GPT-4 on other national exams, there are no studies comparing GPTs to Bard or Bing, so a detailed discussion was unable to be developed. Additionally, although the analysis was conducted using questions from a single national dental hygienist examination, the possibility must be considered that the results may differ from those obtained from multiple years of questions. Furthermore, because the LLMs change as the models are updated, the correct response rate obtained in this study may change over time.

The present study represents the first attempt to use multiple LLMs other than ChatGPT to challenge the Japanese national dental hygienist examination. The best of the four LLMs was shown to be GPT-4, regardless of condition or category. The results of this study indicate that GPT-4 is an LLM that, with an understanding of its limitations, has the potential to be used as an educational support tool for students. However, caution should be exercised in interpreting the results obtained.

## Declaration of competing interest

The authors have no conflicts of interest relevant to this article.
